# Factors of Susceptibility of Human Myiasis Caused by the New World Screw-Worm, *Cochliomyia hominivorax* in São Gonçalo, Rio de Janeiro, Brazil

**DOI:** 10.1673/031.011.0114

**Published:** 2011-02-04

**Authors:** José A. Batista-da-Silva, Gonzalo E. Moya-Borja, Margareth M.C. Queiroz

**Affiliations:** ^1^ Universidade Federal Rural do Rio de Janeiro. Rod. BR 465, Km 7, Seropédica (RJ), Brazil; ^2^ Laboratório de Transmissores de Leishmaniose (Setor de Entomologia Médica e Forense) do Instituto Oswaldo Cruz - IOC/FIOCRUZ, RJ, Brazil

**Keywords:** blowfly, Calliphoridae, parasite, public health, zoophagous

## Abstract

This study was carried out between July 2007 and June 2008 and reports on the occurrence of human myiasis caused by the New World screwworm, *Cochliomyia hominivorax* (Coquerel) (Diptera: Calliphoridae) in São Gonçalo in the state of Rio de Janeiro, Brazil. Liquid or solid vaseline was used to suffocate the larvae, which were then preserved in 70% ethanol and sent to the Instituto Oswaldo Cruz for identification. *C. hominivorax* were identified in all 22 cases of myiasis. There were 12 male and 10 female patients with ages ranging from 03 to 71. Ethnically the highest incidence was among black people, with 17 cases. Open wounds were the main cause of the parasitosis, whereas poor personal hygiene, the low educational level, alcoholism, bedridden patients, and physical or mental disability were possibly secondary factors; in addition to all these factors the income of the patients was very low.

## Introduction

Myiasis is the infestation of living vertebrates by fly larvae that in their immature period, or part of it, feed on the host's dead or living tissue, body substances, or ingested food (Serra-Freire and Mello 2006). They are increasingly common in urban environments and according to Queiroz et al. (2005) they are quite ordinary in rural areas, infesting humans and animals and causing serious economic and public health problems.

The prevalence of infestation by these ectoparasites is an important public health problem and the main predisposing factors associated with this parasite are considered socio-economic. Factors that contribute to the emergence of this parasite include low social condition, alcoholism, mental or neurological diseases, poor personal hygiene, patients with varicose ulcers, diabetes, malnutrition, advanced stages of cancer, pediculosis, immunosuppression, patients with STD, patients with gingivitis, and other lesions in the oral cavity and advanced age (Albernaz 1933; Kaminsky 1993; Verettas et al. 2008). Other factors such as the presence of domestic animals, mendicancy, and unhealthy environments also contribute to the emergence of new cases (Batista-da-Silva et al. 2009).

In humans, in South America, the most frequent species are restricted to *Dermatobia hominis* and *Cochliomyia hominivorax* (Coquerel) (Diptera: Calliphoridae) whose larvae cause obligatory cutaneous myiasis in several mammal (zoophagous) species, including humans. The characteristics of infestation in humans are determined by anatomical, immunological, and pathological factors. Due to their devastating appearance they are also called traumatic myiasis (*C*. *hominivorax)* or furuncular myiasis (*D. hominis*). According to Guimarães and Papavero (1999), the type of infestation can be classified by the characteristics of the larva and the damage that it causes. For this reason, obligate species are those that are parasites on living tissues; and facultative species are those that parasitize necrotic tissues in living individuals. Injuries in the form of boils, with serous or purulent secretion, are characteristics of *D. hominis* infestations. Extensive chronic wounds are usually infested by *C. hominivorax.*

This study investigated the occurrence and epidemiological aspects of human myiasis by *C. hominivorax* in São Gonçalo, an urban area in Rio de Janeiro, Brazil.

## Materials and Methods

The study was carried out between July 2007 and June 2008 with 22 patients treated at the only public hospital in São Gonçalo, RJ that has trained professionals to carry out the collection of fly larvae, to identify and treat myiasis. All the 22 patients with myiasis who attended the outpatient clinic at the public hospital during this period were treated by the medical team that manually removed the larvae.

In order to make the collection of larvae easier, liquid or solid vaseline was used to suffocate the larvae with a dressing over the vaseline and then after one hour the larvae could be removed from the wound more easily, eliminating the use of larvicides (ivermectin). The use of ether, formaldehyde, creolin, tobacco, or sugar was avoided which, although they are effective in killing the larvae and also seen in literature, they are not recommended because they may cause harm to the exposed tissue, allergic reaction, promote bacteria cultures, discomfort, and pain to the patient. All larvae were collected with tweezers, transferred to a container containing 70% ethanol and sent to the Laboratório de Transmissores de Leishmaniose (Setor de Entomologia Médica e Forense) do Instituto Oswaldo Cruz IOC/FIOCRUZ to be identified. In the laboratory, the larvae were clarified, mounted and then identified under a stereoscopic microscope with specific dichotomous keys.

An investigative questionnaire on patient identification, pathological history, social data (physical characteristics of the residence, family income, schooling, profession and alcohol, smoking, and drugs use), practice of recreation and tourist activities was obtained by the health professionals who had been previously trained to recognize the type of myiasis and to collect the fly larvae correctly. After that, the patients signed an informed assent form provided by a professional working at the clinic.

Chi-square (χ^2^) test was performed to determine if the proportions of men and woman parasitized were the same in relation to the patients' ethnic group (white or black), considering the scale for degree of freedom and significance of 5% (p <0.05).

**Table 1.**
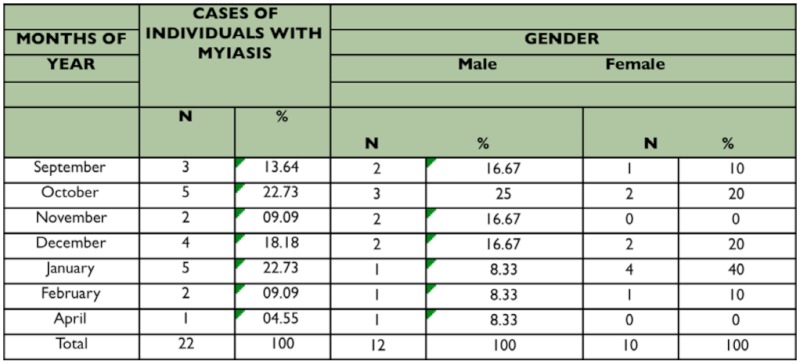
Frequency of human myiasis caused by larvae of *Cochliomyia hominivorax* (Diptera: Calliphoridae), between July 2007 and June 2008.

## Results

Over the twelve month period of this study 22 cases of myiasis among men and women were recorded (Table 1). The larvae were identified using dichotomous keys as belonging to species *C. hominivora.* There were second instars in 9% of the patients and second and third instars in 91% of patients, of which 54.6% were recurrences and 36.4% were first time infections. The larvae of these cases were located as follows: nine (9) on the head; eleven (11) on the legs or feet; one (1) on the abdomen, and one (1) on the back (Table 2). The patients were between 3 and 71 years old (Table 3). The most prevalent age group was from 41 to 50 years old, with equal numbers of infected individuals for black and white ethnic groups. However there was a higher total number of cases among black people (77.27%) (Table 3). This is due mainly to the low level of education (41% of blacks were illiterate and 59% only had elementary school), low hygiene conditions observed (Table 2), and poor urban infrastructure (vacant lots, streets without paving, irregular collection of garbage, and poor distribution of piped water). These facts contribute to a higher incidence of *C. hominivorax.* Another important aspect is the location of patients' homes that was mainly in the São Gonçalo periphery, bordering on rural areas, where infected animals are abandoned frequently (dogs, cats, horses, cattle, and small wild animals).

**Table 2.**
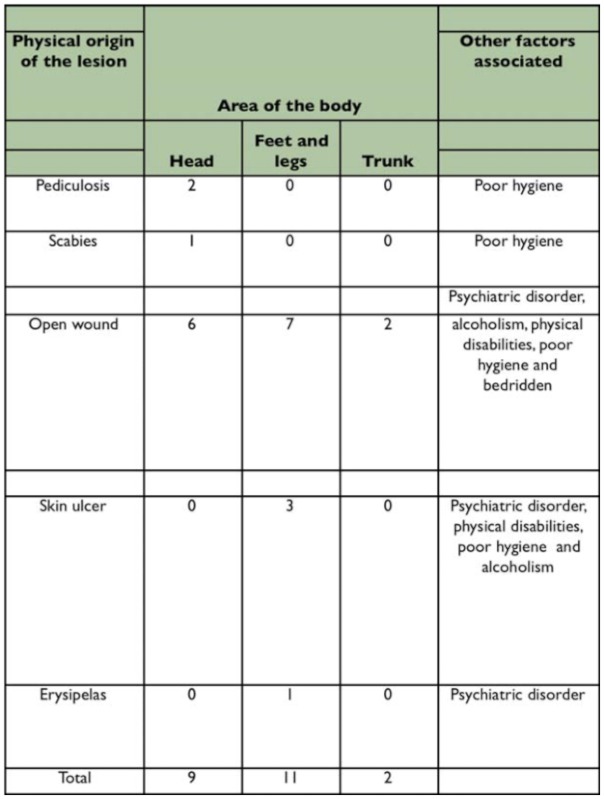
Distribution of human Myiasis cases caused by *Cochliomyia hominivorax* (Diptera: Calliphoridae), according to the origin of the lesion, the area of the body affected and other associated factors.

**Table 3.**
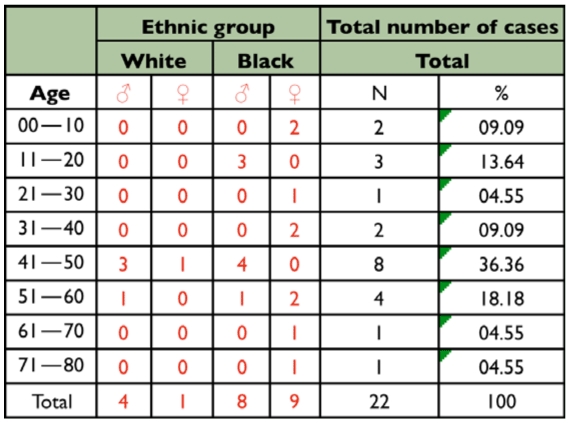
Distribution of the number of cases and the percentage of human myiasis caused by *Cochliomyia hominivorax* (Diptera: Calliphoridae), according to age, gender and ethnic group.

The hottest months of the year indicate a greater occurrence of cases, possibly because people wear light clothes and because the flies find a set of favorable climatic conditions (temperature and humidity) to proliferate. The coldest months show a marked reduction of cases because people use warmer clothes that cover the entire body, and the climatic conditions are not ideal for C. *hominivorax* activity (Table 1).

There was no statistically significant difference between ethnic groups in the proportions of each gender infected ( χ^2^ = 1.68, p> 0.05).

## Discussion

Several species of fly larvae considered obligate or facultative parasites have been reported in different parts of the world by authors such as, Lukin (1989) who recorded 14 cases in 36 months in Brisbane; Kumarasingle et al. (2000) found 16 people parasitized over an 18 month period in hospitals in Sri Lanka; Sherman (2000) identified 42 cases over 36 months in the United States; Oliveira et al. (2004) recorded 68 cases in 12 months in the Baixada Fluminense (RJ); Nascimento et al. (2005) reported 24 cases in 35 months in the city of Recife (PE); Chan et al. (2005) reported 8 cases of human myiasis in 12 months in Hong Kong; Marquez et al. (2007) identified 71 cases in 48 months in three towns in the state of Rio de Janeiro; and Ferraz et al. (2010) recorded a rare case of multiple parasitism with three species of fly larvae causing facultative myiasis in a public hospital in Rio de Janeiro.

Among the main factors of susceptibility mentioned above, Table 2 shows that open wounds were the main cause of the parasitosis whereas poor personal hygiene, the low educational level, alcoholism, bedridden patients, and physical or mental disability were possibly secondary factors that led these people to be infested by *C. hominivorax.* These factors corroborate Lukin's (1989) report which drew attention to the fact that their patients were elderly, sick, and debilitated. Regarding the use of alcohol, Basso (1939) described the insensitivity caused by it - linking it to myiasis. Another important factor was the ethnic group, since 17 (77.27%) of the observed cases occurred in black people and 5 (22.73%) occurred among white people.

There was no significant difference in terms of gender of the parasitized person (Table 1). However according to Marquez et al. (2007), the higher occurrence of myiasis was among men, a fact also observed by Visciarelli et al. (2007) who also described that 76.5% of the cases took place during the summer corroborating the results of this research when the percentage of parasitized males was 54.5%, and emphasizing that most of these men were employed in construction, farming, or domestic activities (75%). Such activities promote greater contact with the flies that cause myiasis. The number of infected women was 45.5%, 90% of whom were black (70% performed housework and 20% were young students), whereas 66.6% of black women were using well water to do their chores and 33.4% used piped and treated water that had an irregular supply (only 1 or 2 times per week) as an aggravating factor confirming the precarious urban infrastructure which described a higher prevalence in the hotter summer months. In this research, the majority of cases of myiasis by *C. hominivorax* were reported as being associated with open wounds confirming the reports of Nascimeto et al. (2005) that reported the presence of preexisting lesions in all cases of myiasis. This can be considered self-violence or neglect of one's health as well as loss of self-esteem due to socio-economic and psychological problems making the patient a temporary displaced person who takes refuge in a hospital until the larvae are removed.

The main areas parasitized were the legs or feet. And those who were parasitized in the head were short in stature, such as children or patients with psychiatric disorders, alcoholism, or the bedridden which shows that the parasites are more common on lower parts of the body. These results confirm those found by Nascimento et al. (2005), who reported in their studies at three public hospitals in Recife that these parts were affected more frequently. Marquez et al. (2007) also described similar data, in his work at three towns in Rio de Janeiro state.

The social, cultural, and financial data demonstrated that the poorest groups with low incomes and low educational levels represented practically all the cases observed; 21 patients received the minimum wage, and only one patient with skin ulcer in the leg was parasitized by the larvae of *C. hominivorax* in the workplace. The patient was a 43 year old, white bar owner where he worked in shorts and light clothes and had an income of five minimum wages. Although this patient had a better socio-economic status, the presence of stray animals such as dogs, cats, horses, and cattle passing near his residence and workplace acted as reservoirs of parasitosis and effective dispersal of larvae of *C. hominivorax* in peri-urban area as described by Batista-da-Silva et al. (2009). Rouquayrol (1994) reported that economically privileged social groups are less exposed to environmental factors that contribute to certain types of diseases compared with the economically less privileged groups. In addition all of the patients in this study had only completed primary education at the most. These data reinforce that ignorance about parasitosis leads the patient to seek medical help late (from the third day of parasitism) causing great pain, discomfort, and reduced productive capacity. Also, besides the low cultural level of all patients observed here, the distance between the patient's residence and the hospital is an additional reason for the delay in seeking the public health service for treatment.

The results presented here reflect a relative expansion of a neglected zoonosis in urban and peri-urban areas where the presence of ills in a modern society such as alcoholism, mendicancy, physical, and mental disabilities and the noticeable difference between ethnic groups that are socially distinct exist. All these factors provide opportunities for a parasite that does not respect the limits of progress, attacking the poorest people and mutilating them in a serious or even permanent way.
